# The Unique Light-Harvesting System of the Algal Phycobilisome: Structure, Assembly Components, and Functions

**DOI:** 10.3390/ijms24119733

**Published:** 2023-06-04

**Authors:** Xiang Li, Wenwen Hou, Jiaxi Lei, Hui Chen, Qiang Wang

**Affiliations:** 1 State Key Laboratory of Crop Stress Adaptation and Improvement, School of Life Sciences, Henan University, Kaifeng 475004, China; lixiangx@mail.ustc.edu.cn (X.L.);; 2 Academy for Advanced Interdisciplinary Studies, Henan University, Kaifeng 475001, China

**Keywords:** phycobilisome, phycobilin, phycobiliprotein, assembly, biosynthesis, photosynthesis

## Abstract

The phycobilisome (PBS) is the major light-harvesting apparatus in cyanobacteria and red algae. It is a large multi-subunit protein complex of several megadaltons that is found on the stromal side of thylakoid membranes in orderly arrays. Chromophore lyases catalyse the thioether bond between apoproteins and phycobilins of PBSs. Depending on the species, composition, spatial assembly, and, especially, the functional tuning of different phycobiliproteins mediated by linker proteins, PBSs can absorb light between 450 and 650 nm, making them efficient and versatile light-harvesting systems. However, basic research and technological innovations are needed, not only to understand their role in photosynthesis but also to realise the potential applications of PBSs. Crucial components including phycobiliproteins, phycobilins, and lyases together make the PBS an efficient light-harvesting system, and these provide a scheme to explore the heterologous synthesis of PBS. Focusing on these topics, this review describes the essential components needed for PBS assembly, the functional basis of PBS photosynthesis, and the applications of phycobiliproteins. Moreover, key technical challenges for heterologous biosynthesis of phycobiliproteins in chassis cells are discussed.

## 1. Introduction

The phycobilisome (PBS) was first identified by Gantt and Conti [1]. PBSs are large chromophore-protein complexes located on the stromal side of thylakoid membranes. As a light-harvesting antenna system, PBSs absorb light between 450 and 650 nm and transmit it efficiently to the reaction centres of the photosynthetic systems of cyanobacteria and red algae.

The main bricks in the spatial arrangement of phycobilisomes are phycobiliproteins (PBPs), which have covalently attached linear open-chain tetrapyrroles called phycobilins, and linker proteins. With the linker proteins, PBPs form the structural backbone of the phycobilisome. Diversity in the number and spatial structure of lyase-catalysed phycobilin binding varies the spectral properties of PBPs. Therefore, phycobilin synthesis, lyase catalysis, and self-assembly of apoproteins and the auxiliary linker proteins are essential for the assembly of phycobiliproteins and the structural and functional stability of the PBS.

The primary prerequisite for photosynthetic light reactions is the capture and efficient transfer of light energy. The unique structure of the phycobilisome determines its function in photosynthesis. The energy of light absorbed by phycobilisomes decreases gradually from the periphery to the core. In this way, it helps to transfer the absorption excitation to the chlorophyll of the reaction centre quickly, efficiently, and unidirectionally. The irreversible transfer of energy from the absorption of light energy by the PBS to photosystem II (PSII) or partially to photosystem I (PSI) may be achieved through the formation of the PBS-PSII-PSI complex. Linker proteins play a key role in the formation of the complex. Moreover, chromatic acclimation (CA) enables PBSs to regulate the pigment composition to optimise light absorption for photosynthesis, thus adapting to fickle light environments [2].

In addition to their role in photosynthesis, PBPs possess excellent spectral properties that can be used in numerous applications. The brightly coloured lustre and fluorescence of the PBP mean that it can be used as a fluorescent probe in bioscience research. Compared with traditional fluorescent probes, PBPs are highly hydrophilic and can maintain their fluorescence properties in aqueous environments for a longer period. Among them, phycoerythrin (PE) is the most stable PBP and is, therefore, the most commonly used PBP fluorescent probe. PBPs are also safe and non-toxic natural products that can be used as a natural colouring for food, cosmetics, and dyes [3].

Phycobilisomes eliminate excess reactive oxygen species, activate antioxidant enzymes, increase antioxidant enzyme activity, and inhibit lipid peroxidation and DNA damage [4]. Furthermore, phycobiliprotein has anti-inflammatory and anticancer effects [5], as well as therapeutic and preventive effects on a wide range of diseases in clinical trials [6]. Based on the antioxidant and optical properties of phycobiliproteins, phycocyanin has been used in various fields such as food, medicine, and cosmetics. Thermophilic cyanobacteria receive a lot of attention because of their extreme heat resistance properties. Their PBS is very important for the adaptation of these cyanobacteria to high-temperature environments. A comparative analysis elucidated distinct molecular components and organization of PBSs in thermophilic cyanobacteria and revealed several highly conserved amino acid sequence motifs of the PBP subunit [7]. PBPs and phycocyanin from extreme environments show great potential as antioxidant, anti-inflammatory, anticancer, antibacterial, antiplatelet, and antiviral compounds [8].

Currently, phycobiliproteins and phycocyanobilin are mainly extracted and purified from *Spirulina* through complex steps. However, the heterologous biosynthesis of PBPs and phycobilins in chassis cells, including *Escherichia coli* and cyanobacteria, has attracted increasing attention. Researchers have achieved the efficient synthesis of phycocyanin in *E. coli* through combinatorial metabolic engineering [9,10]. Some groups have also studied the structure and function of lyase enzymes [11]. Moreover, many investigations have revealed the pharmacological and biological properties of natural or recombinant PBPs, including antioxidant [12], anti-inflammatory [13] and antitumour [14] activities. Synthetic biology and the genetic engineering of chassis cells have enabled the production of various recombinant PBPs, but the rational design of synthetic biological components for the assembly and uniform standardisation of PBPs still faces many challenges.

The structure, function, and applications of PBPs have been extensively studied with particular effort being made on the heterologous biosynthesis and reconstitution of phycobiliproteins. Current hot issues in the PBS field mainly focus on the following aspects: (1) structure and function mining; (2) mechanisms of the excitation energy transfer within the PBS; (3) biosynthesis and dynamic assembly; (4) potential applications, which are critically discussed in this article.

## 2. Requirements for Phycobilisome Assembly

**Figure 1:**
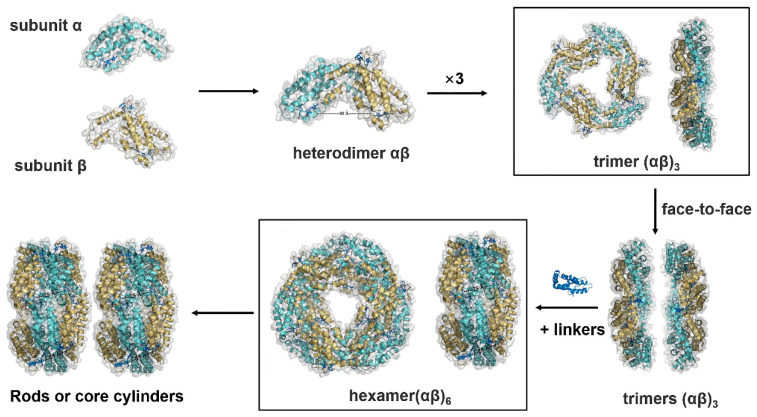
Apoprotein assembly of basic PBS cylinders. α- and β-subunits firstly form a heterodimer αβ as monomers for phycobiliprotein assembly. Three monomers aggregate into the ring-shaped trimer (αβ)_3_ in a head-to-tail manner. Two trimeric disks attach to each other on the α-ring side in a face-to-face manner to form a hexameric (αβ)_6_ disk, which are connected with linker proteins. Such hexamers serve as a basic unit for the assembly of the peripheral rod or core cylinders.

The PBS self-assembles from soluble homologous protein α- and β-subunits of phycobiliproteins that contain conserved cysteine residues to covalently anchor the phycobilins. Both α- and β-subunits have a modified globin fold consisting of eight α-helices. Two additional helices form the association domain between the two subunits in the formation of the heterologous αβ monomer. The assembly process begins with the coupling of phycobilins and apoproteins to form phycobiliprotein subunits, in which the lyase enzyme makes the binding of phycobilins to apoproteins more accurate and catalytically efficient. This process is followed by the spontaneous aggregation of the subunits to form a trimer (αβ)_3_. Next, the trimer binds to the linker proteins, which triggers the formation of the phycobilisome ‘nucleus’ and the binding of the ‘rods’ to form the final phycobilisome [15] (Figure 1).

### 2.1. Phycobiliprotein and PBS Structural Organisation

Crystal structures of PBSs from various cyanobacteria and red algae have been resolved [16,17,18]. PBPs are a group of disk-shaped macromolecular proteins with covalently attached linear open-chain tetrapyrroles known as phycobilins [19]. The basic building block of PBPs is a monomer comprising α- and β-subunits, each with a molecular mass of 15–20 kDa and 160–165 amino acids [15]. The α- and β-subunits are encoded by the genes *cpeA* and *cpeB*, respectively. Each subunit carries heterogeneous phycobilins at conserved cysteine residues. Phycoerythrin subunits mainly use phycoerythrobilin (PEB) or phycourobilin (PUB) that contribute to the 560 nm light absorbance. The heterogeneity of the spectral properties of PBPs enables the PBS to collect excitation energy from the rods and funnel it to the photosynthetic reaction centre. 

The types of PBP based on their pigmentation types can be divided into phycoerythrin (PE), phycoerythrocyanin (PEC), phycocyanin (PC), and allophycocyanin (APC). PE mainly harbours PEB as the major pigmentation to capture the solar energy range from 500–560 nm, while PEC merely appearing in some specific cyanobacteria can combine PVB and PCB. PEC can absorb solar energy in the range of 560–600 nm; PC mainly uses PCB as the main pigment, and mainly captures solar energy in the range of 610–625 nm; APC is also combined with PCB, but the wavelength of absorption is different from PC, and APC mainly absorbs light in the range of 650–660 nm. Noticeably, PE and PEC are not fundamental components for most cyanobacteria PBSs. Meanwhile, most cyanobacteria have PBSs composed exclusively of PC and APC. PE is a relatively rare case and is only found in specific cyanobacteria, such as *Fremyella diplosiphon*.

**Figure 2:**
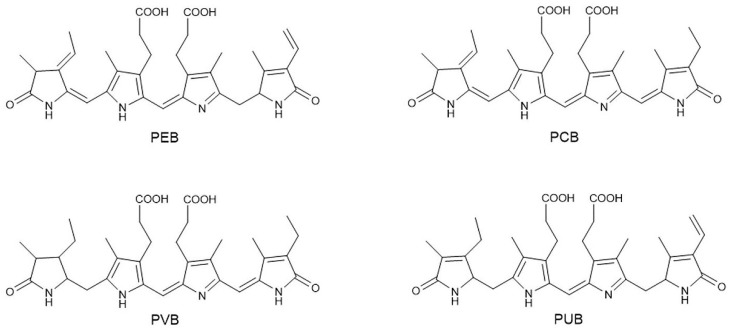
The four main types of phycobilins in algae. Linear open-chain tetrapyrroles make up the main chemical skeleton of phycobilins. These four pigments are isomers, and the position and number of double bonds lead to differences in the spectroscopic properties among phytochromes. PEB—phycoerythrobilin; PCB—phycocyanobilin; PVB—phycoviolobilin; PUB—phycourobilin.

The PBS consists of several peripheral rods that project radially from the central core subcomplex. Typically, the central core consists of a bundle of three core cylinders, each composed of two or four disks of allophycocyanin (APC) trimer [20]. Six rods are bound to the central core and the bundle of core cylinders lies on the surface of the photosystem complexes. The peripheral rods of the PBS supercomplex are made up of phycobiliprotein hexamers together with colourless linker proteins and bilin chromophores [15]. Generally, the rod subcomplex consists of two or more disks of PBP hexamers, typically PE, phycocyanin (PC), or phycoerythrocyanin (PEC). These PBP hexamers are spatially arranged to allow directional energy transfer from PEC or PE to PC within the rod and then to APC in the core [21]. PBP hexamers covalently bind assorted molecules of linear tetrapyrrole chromophores including PEB, phycocyanobilin (PCB), PUB, and phycoviolobilin (PVB) (Figure 2). Phycoerythrin disks associated with PEB or PUB are located in the distal part of the rod and PE absorbs green light of 490–560 nm according to the chromophore composition.

### 2.2. The Synthesis of Phycobilin

**Figure 3:**
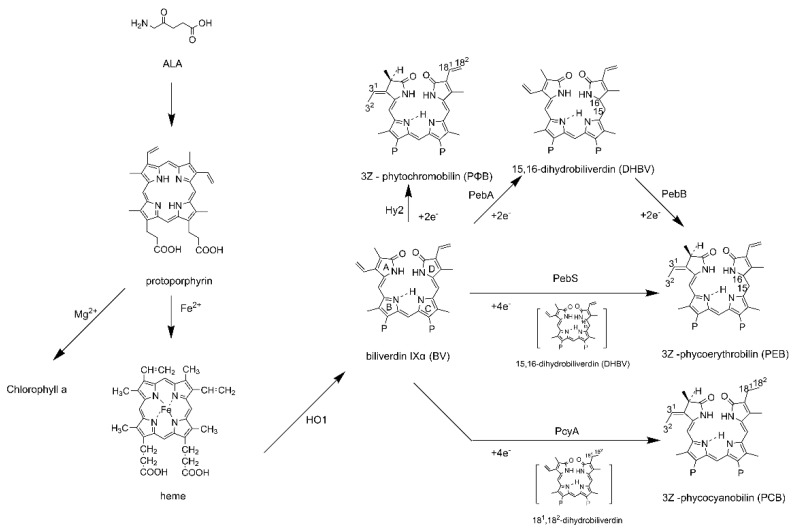
The synthesis pathway of phytochromes. Aminolevulinic acid (ALA) is converted to protoporphyrin. Protoporphyrin chelates Fe^2+^ through the ionic bond to form the haem. Haem oxygenase (HO1) catalyses f haem to the linear and tetrapyrrole biliverdin IXα (BV). BV is further reduced by ferredoxin-dependent bilin reductases (FDBRs) to various light-harvesting chromophores.

In photosynthetic light-harvesting complexes, the main chromophore is the tetrapyrrole derivative. The abundant light-harvesting pigment in plants is chlorophyll, a cyclic tetrapyrrole chromophore. However, in addition to the cyclic tetrapyrrole, there are some open-chain tetrapyrrole chromophores called phycobilins in cyanobacteria. Eight types of phycobilins have been identified [22], of which four (PEB, PCB, PUB, and PVB) are common. The precursor substance of phycobilins is haem, which can be synthesised by haem oxidase (HO) and ferredoxin-dependent bilin reductases (FDBRs) [23]. Aminolevulinic acid (ALA) is converted to protoporphyrin through multistep enzymatic reactions. Protoporphyrin ring chelates Fe^2+^ to produce haem. Subsequently, the haem unravels the closed-chain tetrapyrrole ring, catalysed by haem oxidase, and removes Fe^2+^ to produce the open-chain tetrapyrrole molecule biliverdin IXα (BV), which is then transformed into various phycobilins (in cyanobacteria and red algae) by FDBRs (Figure 3).

Each subunit of PBP can bind one to four phycobilins at conserved cysteine sites [24]. Most PBPs covalently couple with phycobilins through the sulphydryl group of cysteine, generally forming a thioether bond with C31 of the A-ring of PBP. In some phycobiliproteins, C31 of the A-ring and C181 of the D-ring of phycocyanin can form two thioether bonds with two cysteine sulphydryl groups simultaneously [25]. In addition, there are PBP subunits, such as ApcE, that are coupled to PCB through non-covalent bonds [26]. Normally, multiple conserved cysteine sites of PBPs provide anchorage for certain phycobilins. Due to variations in the PBP-bilin pairs, as well as in the spatial arrangements, PBPs exhibit a wide range of spectral properties. Phycocyanin absorbs red light (620–630 nm), PEC absorbs orange light (550–620 nm), and PE absorbs green light (500–560 nm) according to the chromophore composition [21].

**Figure 4:**
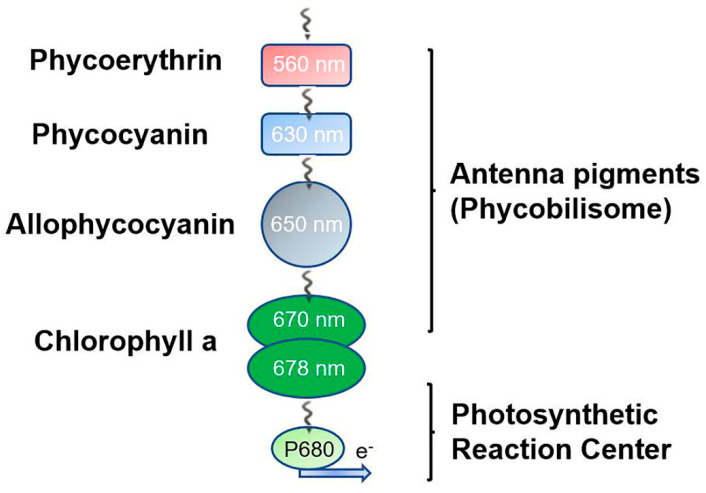
The light energy transfers from peripheral rods and the central core of PBSs to the reaction centre. The solar energy captured by antenna pigments covalently bound to PBPs is delivered to the photosynthetic reaction centre. Then, the energy is transferred irreversibly from rod to core cylinders in one direction. Ultimately, electronic excitation energy is converted to chemical potential with a charge separation process at P680; meanwhile, the released electron funnels into the electron transfer chain.

The light energy absorbed by phycobilins is efficiently transferred among PBPs. Energy is transferred through fluorescence resonance between subunits, then transmitted uniaxially to inferior PBP rods, and finally reaches the reaction centre located in the thylakoid. These PBPs are spatially arranged to allow directional energy transfer from PEC or PE to PC within the rod and then to APC in the core [21]. Chlorophylls capture the energy and transport it to the photosynthetic electron transport chain at an efficiency higher than 95% [27,28] (Figure 4).

### 2.3. Lyases Catalyse the Binding of Phycobilin to Apoproteins

**Figure 5:**
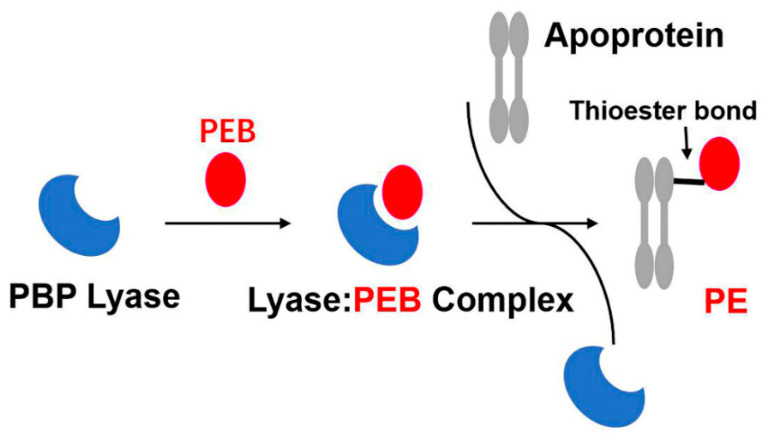
A model for the catalysis of lyases. Particular lyases recognise and combine with corresponding phycobilins to form the lyase—phycobilin complex. Subsequently, the apoprotein (usually the PBP monomer) is activated and integrates a bilin molecule at the conserved cysteine residue via a thioether bond. PBP—phycobiliprotein; PEB—phycoerythrobilin; PE—phycoerythrin.

The attachment of phycobilins to PBPs needs to be catalysed by the corresponding lyase, while some apoproteins can autonomously link with phycobilins at conserved cysteine sites [29], such as the core-membrane linkers (ApcE or L_CM_) that are capable of autocatalytically attaching to PCB [30]. The covalent bond of phycobilins to phycobiliproteins may occur spontaneously both in vivo and in vitro. Nonetheless, specific PBP lyases enable the correct phycobilins to pair with the cysteine residues of PBP subunits appropriately and efficiently (Figure 5).

To date, three major groups of phycobilin lyases have been characterised: S/U-type, T-type, and E/F-type [31,32,33]. The crystal structures of the S/U- and T-type lyases are antiparallel β-barrel structures similar to lipocalin [34,35]. In contrast, the crystal structures of E/F-type lyases adopt a fully helical structure [36].

The first lyase to be discovered that catalyses the covalent linkage of phycocyanin and phycocyanobilin was CpcE/F (CpcE and CpcF co-catalysis). Fairchild et al. (1992) confirmed that CpcE/F catalyses PCB attachment to the cysteine at position 84 of the α-subunit of PC [31,32]. Amino acid homology studies revealed many other types of lyases and that CpcT is homologous to CpeT, which affects the synthesis of PE, and the analysis of PC from CpcT mutants revealed that CpcT can link PC to Cys-153 on the β-subunit of PC [37]. CpeF is a phycoerythrobilin lyase that ligates the double-chain PEB to β-haemoglobin in cyanobacteria [38].

The presence of a chaperone protein is sometimes required for lyase to catalyse the binding of phycobilin to phycobiliprotein. The activity of CpeT is greatly enhanced when the chaperone-like protein CpeZ is present [39].

## 3. Heterologous Biosynthesis of Phycobiliproteins

In the process of heterologous PBP synthesis, the complete pathway can be divided into two steps. The first stage is the synthesis of phycobilins and apoproteins. The phycobilin synthesis is derived from haem, which is first broken down into BV by haem oxygenase, and then BV is reduced to other types of phycobilins by FDBRs. The second stage is the covalent attachment of bilins to apoproteins, a process that is either catalysed by specific lyases or occurs spontaneously [40].

In 1985, the α- and β-subunit genes of allophycocyanin were isolated from the cyanobacteria genome and transferred into *E. coli* to achieve heterologous expression [41]. Since then, many researchers have introduced the *cpcB/A* gene into *E. coli* to explore the recombinant phycobiliprotein subunits [42,43]. The C-phycocyanin β-subunit gene was introduced into *E. coli* and its anticancer effect was studied. The results showed that the β-subunit could inhibit the proliferation of cancer cells and induce cell apoptosis, making it a promising cancer prevention or treatment agent [44]. There have also been many attempts to synthesise phycocyanin in *E. coli* via genetic engineering [9,10]; however, the titre of reconstituted PCB in chassis cells remains low due to the poor catalytic efficiency of the synthetic enzyme and the lack of precursors and cofactors. In a recent study, the synthesis of PCB could be enhanced through assembling haem oxygenase and ferric reductase in appropriate proportions, expressing NAD kinase, and adding NADPH [45]. In addition to heterologous expression in *E. coli*, direct expression of PC in mammalian cells was achieved in 2013 [46].

To obtain complete recombinant PBPs, the phycobilins need to covalently bind with the apoproteins, a process catalysed via self-assembly or lyases. Correct and efficient binding of phycobilins to apoproteins is catalysed by lyases and, therefore, introducing highly efficient lyases into chassis cells is the key to the synthesis of complete PBPs in vivo. E/F-type lyases covalently bind pigments to the α-subunits of phycobiliproteins specifically, and a subclass of E/F-type lyases can chemically modify chromophores [11]. Unlike other lyases, E/F lyase also has chromophore separation activity. CpcS/U specifically binds PCB to Cys-82 of CpcB and Cys-81 of ApcA and ApcB [33]. T-lyase is responsible for attaching pigments to Cys-155 of the phycobilin β-subunit [37,47].

Based on the genetic engineering studies above, it should be possible to achieve large-scale production of low-cost recombinant PBPs and obtain various types of PBPs with improved functions for future applications through molecular design and recombinant synthesis.

## 4. Photosynthetic Function of Phycobilisomes

### 4.1. Light Energy Capture and Transfer in PBS

As a light-harvesting complex, phycobilisomes absorb light energy through pigment molecules and transmit it to the reaction centres of PSII [48]. The structure of an intact PBS in complex with PSII from *Anabaena* sp. strain PCC 7120 was unambiguously resolved using single-particle electron microscopy [49]. Moreover, research focusing on the in situ structures of PBS-PSII-PSI-LHC megacomplexes from the red alga *Porphyridium purpureum* provided interaction details between PBS, PSII, and PSI at near-atomic resolution using cryogenic electron tomography. All these works contribute a solid structural basis for unravelling the mechanisms of the PBS-PSII-PSI-LHC megacomplex assembly, the efficient energy transfer from PBS to the two photosystems, and the regulation of energy distribution between PSII and PSI [50].

Aquatic environments present a unique light environment, and different species of small cyanobacteria and red algae can survive through absorbing a wide range of wavelengths. Phycobilisomes exhibit various and flexible absorption peaks to ensure the efficiency of capturing and transmitting light energy in deep water; in particular, the blue–green light that can penetrate deep water. In addition, PBSs form huge arrays on the thylakoid membranes as antennae. The arrangement of phycobilisomes on the thylakoid membranes is light-intensity-dependent, and their arrangements may be both disordered and ordered. At a light intensity of 15 W·m^−2^ (medium light), the PBSs on the thylakoid membrane are clustered in discrete regions, while at a light intensity of 6 W·m^−2^ (low light), uniformly sized PBSs on the thylakoid membrane are orderly arranged in parallel [51].

Interestingly, cyanobacteria exhibit a form of photomorphogenesis termed chromatic acclimation (CA), and one of the characteristics of CA is the regulation of the pigment composition of PBPs to optimise light absorption for photosynthesis, thus adapting to the light environment in water [2]. The proximity of chromophores in adjacent PBSs suggests that there might also be light energy transfer between PBSs [52].

In the PBS structure, the energy is first received by the PE or PEC at the distal part of the rod and transmitted to the APC core through the PC. Finally, it is transferred to PSII or PSI through the multidomain core–membrane linker (LCM) at the end of the core complex. There are two possible energy transfer pathways in this process: direct energy transfers from PBS to PSI (PBS→PSI transfer) and indirect transfer through PSII (PBS→PSII→PSI transfer) [53]. In terms of the energy level of light absorption, these four PBPs can be further divided into three types: high energy (PE and PEC), medium energy (PC), and low energy (APC). According to the arrangement of PBPs in the PBS, the PBPs form an overall organisation from high to low energy in PBS. Through this holistic organisation, the absorbed excitation energy can be transferred to the auxiliary chlorophyll of the photosystem quickly, efficiently, and directionally. In addition, APC contributes to the excitation of energy from peripheral rods of the PBS or from directly absorbed red light to auxiliary chlorophyll in the photosystem [54].

These large protein complexes capture incident sunlight and transfer the energy to PSII or partially to PSI. This process is achieved through forming PBS-PSII-PSI complexes. Linker proteins play a key role in the formation of modified complexes. ApcE and ApcF are responsible for forming protrusions at the base of the PBS core that fit with the pores on one side of the PSII cell membrane, allowing the PBS and PSII to be tightly connected, which is necessary for the transmission of light energy from PBS to PSII [49]. The abundant aromatic amino acid benzene rings on the linker proteins can also form π–π interactions with the tetrapyrrole rings of surrounding pigment molecules, which are involved in regulating the energy state of the pigment molecules to ensure efficient unidirectional energy transfer [16].

### 4.2. Light Acclimation of PBS

The structure and specialised function of the PBS allows captured light energy to be transferred to the photosynthetic reaction centres with more than 95% efficiency [27]. However, excessive light harvesting can also cause damage to cyanobacteria, so cyanobacteria have evolved a photoprotection mechanism called non-photochemical quenching (NPQ) that rapidly converts excess excited energy into heat before it causes damage. However, this process leads to a reduction in the efficiency of light energy conversion. A photoprotection mode is mediated by orange carotenoid protein (OCP), which is the only known carotenoid-activated photoreceptor, and only exists in cyanobacteria to play a role in controlling the photosynthetic mechanism of light capture. It is known to change from OCP^O^ to OCP^R^ after absorbing redundant blue–green light, and then four OCP^R^ form two dimers which are bound to PBS, respectively, leading to NPQ. Notably, not every PBS is equally sensitive to NPQ, and only one of the three PBS conformational states reported associations with the OCP^R^ [17]. The difference in the conformational state is generated through switching the position of the two rods, which regulates light harvesting.

To adapt to changes in environmental conditions, the composition and function of PBSs would change accordingly. Light intensity has the greatest influence on the composition of the rod and the ratio of PC:APC so as to yield a maximum production of PC under optimal photon flux. Other environmental factors also change the composition of the rods. For example, light colour and temperature can change the PC:PE ratio [55]. The photosynthetic electron transfer rate of PSII also affects the structure of the PBS. The ratio of PBS to chlorophyll protein content is influenced by the electron transfer rate when the required phytochrome is sufficient. Changes in copper ion concentration affect the stability of PBS, and changes in the structural stability of PBS follow the same trend as changes in the rate of electron transfer associated with copper ion concentration, and the structural stability of PBS decreases with the decrease in electron transfer rate, which may be related to structural changes in the rods. Iron deficiency inhibits PBS synthesis but does not affect the stability of PBS.

Degradation of PBS plays an important role in photoprotection, cell maintenance, growth, and development in a constantly changing environment. During nitrogen limitation, PBSs in cells are degraded to avoid excessive light uptake and to allocate effective nitrogen to functions essential for growth and survival [56]. As a large nutrient reserve, the degradation of PBSs can provide essential amino acids for metabolic processes, and low levels of photosynthesis and loss of pigments are essential for cell survival during nitrogen, sulphur, or phosphorus depletion. NblB and NblA are essential components for phycocyanin degradation under starvation conditions, during which NblB levels decrease approximately twofold and directly mediate pigment degradation through chromophore segregation. In contrast, NblA is highly expressed during starvation and may bind to one or two PBS complexes, destabilising the PBS complex and initiating proteochrome degradation [57]. NblB-dependent PC degradation did not occur in the absence of NblA, so NblB has a dependent effect on NblA [58]. Recent studies have found that NblD plays a critical role in the coordinated catabolism of PBSs and, thus, is a factor in the genetically programmed response to nitrogen starvation [59].

## 5. Prospects

In recent decades, we have achieved great progress in our understanding of the PBS, a unique light-harvesting system in cyanobacteria and red algae. Different from the LHC of eukaryotic plants, PBSs possess a more sophisticated structure and a more flexible combination of chromophores, which enables PBPs to capture a wide wavelength of solar energy (including 500–560 nm green light absorbed by PE in particular red algae) and funnel it into the photosynthetic reaction centre. As a light-harvesting complex of cyanobacteria and red algae, phycobilisomes have excellent and unique functional properties. Phycobilisomes are rich in pigment molecules and cover a wide spectrum of absorption, absorbing light wavelengths of 450–650 nm and even more than 700 nm in some cases, compensating for the reduced absorption of chlorophyll at 500–600 nm. PBS has a very high efficiency of transferring energy to the photoreaction centre. It can transfer the captured light energy to the photoreaction centre with an efficiency of more than 95%, which has a lot to do with the special structure and spatial arrangement of phycobilisomes. It allows cyanobacteria and red algae to survive in low light or different light colours. Moreover, cyanobacteria evolved a photoprotection mechanism. After absorbing blue and green light, the resting carotenoid molecule OCP^O^ would be transformed into OCP^R^, which combined with PBS and led to NPQ, ensuring that cells would not be damaged under strong light. In addition, the protein composition of PBSs can change with the light intensity, temperature, and other environmental factors, and the size of PBSs can also become larger or smaller according to the needs of organisms. In conclusion, as the light-catching complex of cyanobacteria and red algae, PBSs possess unique pigment molecules and energy transfer mechanisms different from common eukaryotic green plants, which enables organisms with this structure to adapt to the environment well and survive in such a complex and changeable environment in a good state. With multiple in-depth studies on the components for PBS assembly, it should be possible to simulate the assembly and energy capture–delivery route in the future. Noticeably, synthetic biology, integrated structural biology, genetic engineering, and biochemistry provide a rational design of the PBP’s reconstruction in chassis cells such as *E. coli*. To date, fluorescent β-subunits of C-phycocyanin from *Spirulina* have achieved significant biosynthetic production in engineered *E. coli* [43]. However, the structural organisation between various PBP rods and the assembly for intact PBSs with fine-tuned functions remain unclear. Therefore, standardized biological elements designed via synthetic biology are urgently needed for PBS reconstitution in chassis cells. More in-depth studies on heterologous reconstitution of fully functional PBSs would help not only to reveal the process of PBP self-assembly to form well-organised PBS rods but also to illustrate their light-harvesting and energy-delivery capabilities.

With the development of synthetic biology research, more rationally designed biological components including PBPs, linker proteins, and chromophore lyases would be identified. Through the construction of genetically engineered chassis cells (*E. coli* or cyanobacteria), producing reconstituted PBPs, including recombinant PC or PE, at a large scale would be possible, thus broadening the scope of their application. However, the scheme and application of artificial light-harvesting apparatus still face multiple technical challenges. Many attempts have been made to reconstitute the α- or β-subunit of PBPs, particularly those of phycocyanin, whereas how these subunits are organized and assembled onto light-harvesting antennae remains obscure due to the intricate interaction among PBPs. Although structural and synthetic biology gave us a structural basis of PBSs and a pathway for recombinant PBPs, investigations on the dynamic assembly and energy transfer process of PBS rods progress slowly. Therefore, it is of great significance to apply synthetic biology for building photosynthetic antennae components in order to uncover the dynamic assembly and energy-delivery route among PBPs. Meanwhile, the heterologous reconstitution of specific PBS rods would expand the capability of photosynthetic light-harvesting systems to broaden the absorption wavelength and utilize solar energy. For example, the biosynthesis and assembly of PE into the light-harvesting apparatus of other species may expand the range of wavelength absorbance and improve the efficiency of light-trapping devices under unique light conditions. The highly efficient utilisation of solar energy under low light or diverse light quality might be accelerated through establishing artificial light-harvesting antennae. More importantly, the establishment of uniform and standardized photosynthetic antennae elements through synthetic biology will greatly expand the boundaries of solar energy absorption for different species and significantly improve the efficiency of natural light utilization.

## Data Availability

No data was used for the research described in the article.

## References

[1] Gantt E., Conti S.F. (1966). Phycobiliprotein localization in algae. Brookhaven Symp. Biol..

[2] Montgomery B.L. (2017). Seeing new light: Recent insights into the occurrence and regulation of chromatic acclimation in cyanobacteria. Curr. Opin. Plant. Biol..

[3] Mysliwa-Kurdziel B., Solymosi K. (2017). Phycobilins and Phycobiliproteins Used in Food Industry and Medicine. Mini Rev. Med. Chem..

[4] Wu Q., Liu L., Miron A., Klímová B., Wan D., Kuča K. (2016). The antioxidant, immunomodulatory, and anti-inflammatory activities of Spirulina: An overview. Arch. Toxicol..

[5] Leung P.O., Lee H.H., Kung Y.C., Tsai M.F., Chou T.C. (2013). Therapeutic effect of C-phycocyanin extracted from blue green algae in a rat model of acute lung injury induced by lipopolysaccharide. Evid. Based Complement. Altern. Med..

[6] Jiang L., Wang Y., Liu G., Liu H., Zhu F., Ji H., Li B. (2018). C-Phycocyanin exerts anti-cancer effects via the MAPK signaling pathway in MDA-MB-231 cells. Cancer Cell Int..

[7] Tang J., Zhou H., Yao D., Du L., Daroch M. (2023). Characterization of Molecular Diversity and Organization of Phycobilisomes in Thermophilic Cyanobacteria. Int. J. Mol. Sci..

[8] Puzorjov A., McCormick A.J. (2020). Phycobiliproteins from extreme environments and their potential applications. J. Exp. Bot..

[9] Mukougawa K., Kanamoto H., Kobayashi T., Yokota A., Kohchi T. (2006). Metabolic engineering to produce phytochromes with phytochromobilin, phycocyanobilin, or phycoerythrobilin chromophore in *Escherichia coli*. FEBS Lett..

[10] Ge B., Li Y., Sun H., Zhang S., Hu P., Qin S., Huang F. (2013). Combinational biosynthesis of phycocyanobilin using genetically-engineered *Escherichia coli*. Biotechnol. Lett..

[11] Zhao C., Höppner A., Xu Q.Z., Gärtner W., Scheer H., Zhou M., Zhao K.H. (2017). Structures and enzymatic mechanisms of phycobiliprotein lyases CpcE/F and PecE/F. Proc. Natl. Acad. Sci. USA.

[12] Nakagawa K., Ritcharoen W., Sri-Uam P., Pavasant P., Adachi S. (2016). Antioxidant properties of convective-air-dried Spirulina maxima: Evaluation of phycocyanin retention by a simple mathematical model of air-drying. Food Bioprod. Process..

[13] Liu Q., Wang Y., Cao M., Pan T., Yang Y., Mao H., Sun L., Liu G. (2015). Anti-allergic activity of R-phycocyanin from Porphyra haitanensis in antigen-sensitized mice and mast cells. Int. Immunopharmacol..

[14] Deniz I., Ozen M.O., Yesil-Celiktas O. (2016). Supercritical fluid extraction of phycocyanin and investigation of cytotoxicity on human lung cancer cells. J. Supercrit. Fluids.

[15] Li W., Su H.N., Pu Y., Chen J., Liu L.N., Liu Q., Qin S. (2019). Phycobiliproteins: Molecular structure, production, applications, and prospects. Biotechnol. Adv..

[16] Ma J., You X., Sun S., Wang X., Qin S., Sui S.F. (2020). Structural basis of energy transfer in Porphyridium purpureum phycobilisome. Nature.

[17] Domínguez-Martín M.A., Sauer P.V., Kirst H., Sutter M., Bína D., Greber B.J., Nogales E., Polívka T., Kerfeld C.A. (2022). Structures of a phycobilisome in light-harvesting and photoprotected states. Nature.

[18] Kawakami K., Hamaguchi T., Hirose Y., Kosumi D., Miyata M., Kamiya N., Yonekura K. (2022). Core and rod structures of a thermophilic cyanobacterial light-harvesting phycobilisome. Nat. Commun..

[19] Apt K.E., Collier J.L., Grossman A.R. (1995). Evolution of the phycobiliproteins. J. Mol. Biol..

[20] Adir N., Bar-Zvi S., Harris D. (2020). The amazing phycobilisome. Biochim. Biophys. Acta Bioenerg..

[21] Watanabe M., Ikeuchi M. (2013). Phycobilisome: Architecture of a light-harvesting supercomplex. Photosynth. Res..

[22] Zhou J., Gasparich G.E., Stirewalt V.L., de Lorimier R., Bryant D.A. (1992). The cpcE and cpcF genes of *Synechococcus* sp. PCC 7002. Construction and phenotypic characterization of interposon mutants. J. Biol. Chem..

[23] Dammeyer T., Frankenberg-Dinkel N. (2008). Function and distribution of bilin biosynthesis enzymes in photosynthetic organisms. Photochem. Photobiol. Sci..

[24] Blot N., Wu X.J., Thomas J.C., Zhang J., Garczarek L., Böhm S., Tu J.M., Zhou M., Plöscher M., Eichacker L. (2009). Phycourobilin in trichromatic phycocyanin from oceanic cyanobacteria is formed post-translationally by a phycoerythrobilin lyase-isomerase. J. Biol. Chem..

[25] Sonani R.R., Roszak A.W., Ortmann de Percin Northumberland C., Madamwar D., Cogdell R.J. (2018). An improved crystal structure of C-phycoerythrin from the marine cyanobacterium *Phormidium* sp. A09DM. Photosynth. Res..

[26] Miao D., Ding W.L., Zhao B.Q., Lu L., Xu Q.Z., Scheer H., Zhao K.H. (2016). Adapting photosynthesis to the near-infrared: Non-covalent binding of phycocyanobilin provides an extreme spectral red-shift to phycobilisome core-membrane linker from *Synechococcus* sp. PCC7335. Biochim. Biophys. Acta.

[27] Zhang Z., Lambrev P.H., Wells K.L., Garab G., Tan H.S. (2015). Direct observation of multistep energy transfer in LHCII with fifth-order 3D electronic spectroscopy. Nat. Commun..

[28] Sidler W.A., Bryant D.A. (1994). Phycobilisome and Phycobiliprotein Structures. The Molecular Biology of Cyanobacteria.

[29] Zolla L., Bianchetti M., Rinalducci S. (2002). Functional studies of the *Synechocystis* phycobilisomes organization by high performance liquid chromatography on line with a mass spectrometer. Eur. J. Biochem..

[30] Zhao K.H., Su P., Böhm S., Song B., Zhou M., Bubenzer C., Scheer H. (2005). Reconstitution of phycobilisome core-membrane linker, LCM, by autocatalytic chromophore binding to ApcE. Biochim. Biophys. Acta.

[31] Fairchild C.D., Zhao J., Zhou J., Colson S.E., Bryant D.A., Glazer A.N. (1992). Phycocyanin alpha-subunit phycocyanobilin lyase. Proc. Natl. Acad. Sci. USA.

[32] Fairchild C.D., Glazer A.N. (1994). Oligomeric structure, enzyme kinetics, and substrate specificity of the phycocyanin alpha subunit phycocyanobilin lyase. J. Biol. Chem..

[33] Saunée N.A., Williams S.R., Bryant D.A., Schluchter W.M. (2008). Biogenesis of phycobiliproteins: II. CpcS-I and CpcU comprise the heterodimeric bilin lyase that attaches phycocyanobilin to CYS-82 of beta-phycocyanin and CYS-81 of allophycocyanin subunits in *Synechococcus* sp. PCC 7002. J. Biol. Chem..

[34] Kronfel C.M., Kuzin A.P., Forouhar F., Biswas A., Su M., Lew S., Seetharaman J., Xiao R., Everett J.K., Ma L.C. (2013). Structural and biochemical characterization of the bilin lyase CpcS from Thermosynechococcus elongatus. Biochemistry.

[35] Overkamp K.E., Gasper R., Kock K., Herrmann C., Hofmann E., Frankenberg-Dinkel N. (2014). Insights into the biosynthesis and assembly of cryptophycean phycobiliproteins. J. Biol. Chem..

[36] Kumarapperuma I., Joseph K.L., Wang C., Biju L.M., Tom I.P., Weaver K.D., Grébert T., Partensky F., Schluchter W.M., Yang X. (2022). Crystal structure and molecular mechanism of an E/F type bilin lyase-isomerase. Structure.

[37] Shen G., Saunée N.A., Williams S.R., Gallo E.F., Schluchter W.M., Bryant D.A. (2006). Identification and characterization of a new class of bilin lyase: The cpcT gene encodes a bilin lyase responsible for attachment of phycocyanobilin to Cys-153 on the beta-subunit of phycocyanin in *Synechococcus* sp. PCC 7002. J. Biol. Chem..

[38] Kronfel C.M., Hernandez C.V., Frick J.P., Hernandez L.S., Gutu A., Karty J.A., Boutaghou M.N., Kehoe D.M., Cole R.B., Schluchter W.M. (2019). CpeF is the bilin lyase that ligates the doubly linked phycoerythrobilin on β-phycoerythrin in the cyanobacterium Fremyella diplosiphon. J. Biol. Chem..

[39] Nguyen A.A., Joseph K.L., Bussell A.N., Pokhrel S., Karty J.A., Kronfel C.M., Kehoe D.M., Schluchter W.M. (2020). CpeT is the phycoerythrobilin lyase for Cys-165 on β-phycoerythrin from Fremyella diplosiphon and the chaperone-like protein CpeZ greatly improves its activity. Biochim. Biophys. Acta Bioenerg..

[40] Biswas A., Vasquez Y.M., Dragomani T.M., Kronfel M.L., Williams S.R., Alvey R.M., Bryant D.A., Schluchter W.M. (2010). Biosynthesis of cyanobacterial phycobiliproteins in *Escherichia coli*: Chromophorylation efficiency and specificity of all bilin lyases from *Synechococcus* sp. strain PCC 7002. Appl. Environ. Microbiol..

[41] Bryant D.A., de Lorimier R., Lambert D.H., Dubbs J.M., Stirewalt V.L., Stevens S.E., Porter R.D., Tam J., Jay E. (1985). Molecular cloning and nucleotide sequence of the alpha and beta subunits of allophycocyanin from the cyanelle genome of Cyanophora paradoxa. Proc. Natl. Acad. Sci. USA.

[42] Yu P., Li P., Chen X., Chao X. (2016). Combinatorial biosynthesis of *Synechocystis* PCC6803 phycocyanin holo-α-subunit (CpcA) in *Escherichia coli* and its activities. Appl. Microbiol. Biotechnol..

[43] Wu X.J., Yang H., Chen Y.T., Li P.P. (2018). Biosynthesis of Fluorescent β Subunits of C-Phycocyanin from *Spirulina subsalsa* in *Escherichia coli*, and Their Antioxidant Properties. Molecules.

[44] Wang H., Liu Y., Gao X., Carter C.L., Liu Z.R. (2007). The recombinant beta subunit of C-phycocyanin inhibits cell proliferation and induces apoptosis. Cancer Lett..

[45] Zhao X., Gao H., Wang Y., Wang Z., Zhou J. (2022). Efficient Synthesis of Phycocyanobilin by Combinatorial Metabolic Engineering in *Escherichia coli*. ACS Synth. Biol..

[46] Müller K., Engesser R., Timmer J., Nagy F., Zurbriggen M.D., Weber W. (2013). Synthesis of phycocyanobilin in mammalian cells. Chem. Commun..

[47] Zhou W., Ding W.L., Zeng X.L., Dong L.L., Zhao B., Zhou M., Scheer H., Zhao K.H., Yang X. (2014). Structure and mechanism of the phycobiliprotein lyase CpcT. J. Biol. Chem..

[48] Sui S.F. (2021). Structure of Phycobilisomes. Annu. Rev. Biophys..

[49] Chang L., Liu X., Li Y., Liu C.C., Yang F., Zhao J., Sui S.F. (2015). Structural organization of an intact phycobilisome and its association with photosystem II. Cell Res..

[50] You X., Zhang X., Cheng J., Xiao Y., Ma J., Sun S., Zhang X., Wang H.W., Sui S.F. (2023). In situ structure of the red algal phycobilisome-PSII-PSI-LHC megacomplex. Nature.

[51] Folea I.M., Zhang P., Aro E.M., Boekema E.J. (2008). Domain organization of photosystem II in membranes of the cyanobacterium *Synechocystis* PCC6803 investigated by electron microscopy. FEBS Lett..

[52] Zheng L., Zheng Z., Li X., Wang G., Zhang K., Wei P., Zhao J., Gao N. (2021). Structural insight into the mechanism of energy transfer in cyanobacterial phycobilisomes. Nat. Commun..

[53] Ueno Y., Aikawa S., Niwa K., Abe T., Murakami A., Kondo A., Akimoto S. (2017). Variety in excitation energy transfer processes from phycobilisomes to photosystems I and II. Photosynth. Res..

[54] Soulier N., Bryant D.A. (2021). The structural basis of far-red light absorbance by allophycocyanins. Photosynth. Res..

[55] Chenu A., Keren N., Paltiel Y., Nevo R., Reich Z., Cao J. (2017). Light Adaptation in Phycobilisome Antennas: Influence on the Rod Length and Structural Arrangement. J. Phys. Chem. B.

[56] Yoshihara A., Kobayashi K. (2022). Photosynthesis and Cell Growth Trigger Degradation of Phycobilisomes during Nitrogen Limitation. Plant Cell Physiol..

[57] Nagarajan A., Zhou M., Nguyen A.Y., Liberton M., Kedia K., Shi T., Piehowski P., Shukla A., Fillmore T.L., Nicora C. (2019). Proteomic Insights into Phycobilisome Degradation, A Selective and Tightly Controlled Process in The Fast-Growing Cyanobacterium *Synechococcus* elongatus UTEX 2973. Biomolecules.

[58] Levi M., Sendersky E., Schwarz R. (2018). Decomposition of cyanobacterial light harvesting complexes: NblA-dependent role of the bilin lyase homolog NblB. Plant J..

[59] Krauspe V., Fahrner M., Spät P., Steglich C., Frankenberg-Dinkel N., Maček B., Schilling O., Hess W.R. (2021). Discovery of a small protein factor involved in the coordinated degradation of phycobilisomes in cyanobacteria. Proc. Natl. Acad. Sci. USA.

